# Supplementary material for: Design, Synthesis, and Biological Evaluation of N-Acylhydrazones and Their Activity Against Leishmania amazonensis Promastigotes
Source: Microorganisms. 2025 Jul 2;13(7):1563. doi: 10.3390/microorganisms13071563 (PMC12298860; doi:10.3390/microorganisms13071563)
Supplement: Supplementary file 1 [file microorganisms-13-01563-s001.zip › HPLC amostras-20241114T144635Z-001.zip!/HPLC amostras/HPLC composto 36 - PMM 07 - 18.pdf]

# Farmanguinhos - Fiocruz
# Serviço de Métodos Analíticos

PMM 07 - 18.lcd
2018 - Sintese 1 - João Mafra - 2-5/2-84-4 - PMM 07 - 18.lcd
21/06/2018, 23:37:50

| | | | |
|---|---|---|---|
| Sample Name | : PMM 07 - 18 | | |
| Sample ID | : 118747 | | |
| Sample Full Path | : 2018 - Sintese 1 - João Mafra - 2-5/2-84-4 - PMM 07 - 18.lcd | | |
| Data Filename | : PMM 07 - 18.lcd | | |
| Method Filename | : Nitroimidazol.lcm | | |
| Batch File | : 2018 - Sintese 1 - João Mafra - CLAE-10 - CLAE 10_2018 _0619.lcb | | |
| Vial # | : 1-46 | Sample Type | : Unknown |
| Injection Volume | : 10 uL | | |
| Date Acquired | : 21/06/2018 23:38:19 | Acquired by | : Marcio Soares |
| Date Processed | : 27/06/2018 08:57:30 | Processed by | : Marcio Soares |
| Data Comment | : Método: Nitroimidazol | | |

Preparo da Amostra: 1,08 mg foram adicionados 4 mL de metanol, agitada em vortex e levada ao ultrassom por 3 min. Posteriormente filtrada em membrana PVDF de 0,22µm e injetada.

## Method

<<Header>>

<<Pump>>
Total Flow : 1,0000 mL/min
Pump A : LC-20ADXR

<<Mobile Phase Name>>
Pump A Mobile Phase A : Água
Pump A Mobile Phase C : Acetonitrila

<<LC Time Program>>

| Time | Module | Command | Value | C |
|---|---|---|---|---|
| 0,01 | Pumps | Solvent C Conc. | 20 | |
| 0,01 | Pumps | Total Pump A Flow | 1 | |
| 5,00 | Pumps | Solvent C Conc. | 20 | |
| 22,00 | Pumps | Solvent C Conc. | 90 | |
| 25,00 | Pumps | Solvent C Conc. | 90 | |
| 33,00 | Pumps | Solvent C Conc. | 20 | |
| 39,00 | Pumps | Solvent C Conc. | 20 | |
| 39,01 | Controller | Stop | | |

<<Data Acquisition>>
LC Stop Time : 39,01 min

<<Oven>>
Oven Temperature : 30 C

## Instrumentation

<<Instrument>>

<<Pump A>>
Pump A Type : LC-20ADXR

<<System Controller>>
SCL Type : CBM-20A

<<Autosampler>>
Autosampler : SIL-30AC

<<Oven>>
Oven Type : CTO-20AC

<<Column>>
Column Name : Supelcosil LC- 8 - 5 µm
Column ID : CL 0173
Length : 150 mm
Internal Diameter : 4,6 mm
Max Number of Injections : 9999
Expiration Date : 28/04/2015
Max Temperature : 80,0 C
Installation Date : 28/04/2030

<<PDA>>
Detector Name : PDA
Detector Type : SPD-M20A

<<Degasser>>
Degasser : DGU-20A5R

# Farmanguinhos - Fiocruz
# Serviço de Métodos Analíticos

PMM 07 - 18.lcd
2018 - Sintese 1 - João Mafra - 2-5/2-84-4 - PMM 07 - 18.lcd
21/06/2018, 23:37:50

Chromatogram

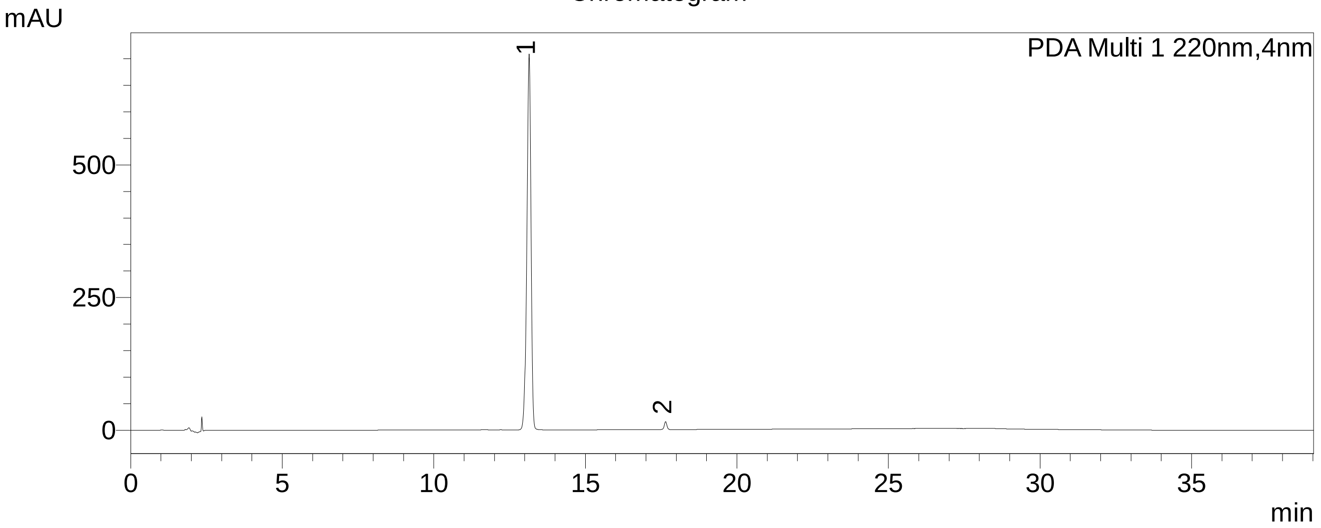


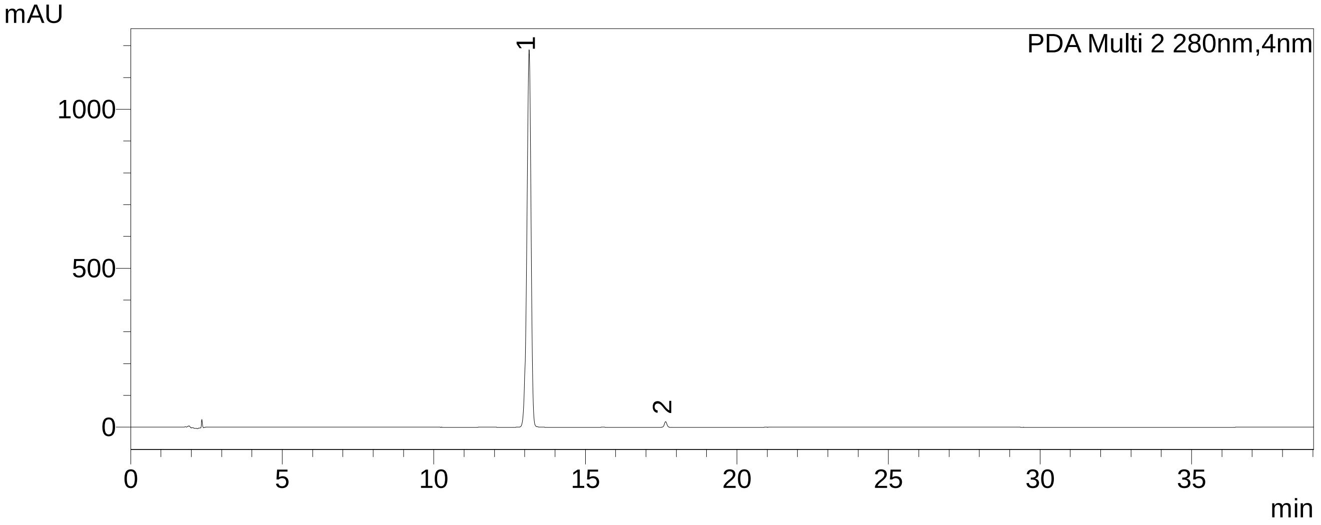


Peak Table

PDA Ch1 220nm

| Peak# | Ret. Time | Name | Area | Area% | Theoretical Plates/meter(USP) | Tailing Factor | Resolution(USP) | Capacity Factor(k') |
|---|---|---|---|---|---|---|---|---|
| 1 | 13,14 | | 6683583 | 98,7 | 267091 | 0,912 | -- | -- |
| 2 | 17,64 | | 89784 | 1,3 | 1067467 | 1,013 | 20,518 | 0,343 |
| Total | | | 6773366 | 100,0 | | | | |

PDA Ch2 280nm

| Peak# | Ret. Time | Name | Area | Area% | Theoretical Plates/meter(USP) | Tailing Factor | Resolution(USP) | Capacity Factor(k') |
|---|---|---|---|---|---|---|---|---|
| 1 | 13,14 | | 11322394 | 99,1 | 262356 | 0,911 | -- | -- |
| 2 | 17,65 | | 108281 | 0,9 | 1067300 | 1,013 | 20,407 | 0,343 |
| Total | | | 11430675 | 100,0 | | | | |
